# CTTGAN: Traffic Data Synthesizing Scheme Based on Conditional GAN

**DOI:** 10.3390/s22145243

**Published:** 2022-07-13

**Authors:** Jiayu Wang, Xuehu Yan, Lintao Liu, Longlong Li, Yongqiang Yu

**Affiliations:** 1 College of Electronic Engineering, National University of Defense Technology, Hefei 230037, China; wangjiayu@nudt.edu.cn (J.W.); lintao89@nudt.edu.cn (L.L.); lilongs@nudt.edu.cn (L.L.); publicmouse@126.com (Y.Y.); 2 Anhui Province Key Laboratory of Cyberspace Security Situation Awareness and Evaluation, Hefei 230037, China

**Keywords:** malicious traffic identification, conditional GAN, sample synthesis, data balancing

## Abstract

Most machine learning algorithms only have a good recognition rate on balanced datasets. However, in the field of malicious traffic identification, benign traffic on the network is far greater than malicious traffic, and the network traffic dataset is imbalanced, which makes the algorithm have a low identification rate for small categories of malicious traffic samples. This paper presents a traffic sample synthesizing model named Conditional Tabular Traffic Generative Adversarial Network (CTTGAN), which uses a Conditional Tabular Generative Adversarial Network (CTGAN) algorithm to expand the small category traffic samples and balance the dataset in order to improve the malicious traffic identification rate. The CTTGAN model expands and recognizes feature data, which meets the requirements of a machine learning algorithm for training and prediction data. The contributions of this paper are as follows: first, the small category samples are expanded and the traffic dataset is balanced; second, the storage cost and computational complexity are reduced compared to models using image data; third, discrete variables and continuous variables in traffic feature data are processed at the same time, and the data distribution is described well. The experimental results show that the recognition rate of the expanded samples is more than 0.99 in MLP, KNN and SVM algorithms. In addition, the recognition rate of the proposed CTTGAN model is better than the oversampling and undersampling schemes.

## 1. Introduction

The rapid development of network technology brings convenience to people. However, it is also accompanied by security problems. Many devices that meet technical requirements can access the Internet, including software with malicious behaviors, such as invading users’ hosts, stealing information, destroying equipment, etc., bringing great hidden dangers to users’ privacy and to the security of their property. The secure protection of information and property on the network is a key problem to be solved, and the accurate identification of traffic plays an important role in solving this problem.

The methods of malicious traffic identification mainly include port-based [1], payload-based [2,3] and machine learning algorithms. The port based identification method is no longer suitable for the current network environment because many network attacks no longer use fixed and conventional ports. However, identification methods based on payloads cannot identify the encrypted traffic, and at present, traffic encryption in the network has become a gradual trend. In order to solve the problem of encrypted malicious traffic identification, people began to study the identification method based on machine learning algorithms.

Lucia et al. [4] used Convolutional Neural Networks (CNN) and Support Vector Machine (SVM) algorithms to identify traffic, and the recognition effect of SVM algorithm was better than the CNN. Shekhawat et al. [5] used three machine learning algorithms (SVM, XGBoost, Random Forest) to identify traffic respectively, and further analyzed the extracted features. They suggested that feature selection based on the model itself (domain free method) may be better than selecting features based on human expertise. Some researchers use deep learning algorithms to automatically extract features and then identify traffic [6,7,8]. He et al. [9] proposed a malicious traffic detection method based on a CNN and Auto-Encoders (AE). The encoder is trained with benign traffic to learn its reconstruction ability. When malicious traffic is input into the encoder, the reconstruction rate cannot reach the threshold; that is, the traffic is judged as a malicious one. Zhong et al. [10] proposed a heterogeneous ensemble-learning traffic detection framework based on multiple deep learning models.

Many machine learning algorithms work well on balanced datasets but not on imbalanced datasets. In the real network environment, benign traffic has a large amount of data and is easy to collect, while malicious traffic has a small amount of data and is difficult to collect. In many network traffic datasets, benign traffic is far more than malicious traffic. Similar situations exist in practical applications. We need to accurately identify malicious traffic in a large number of benign traffic. Data imbalance leads to the low recognition accuracy of many machine learning models. People have conducted a lot of research to solve this problem. From the perspective of model improvement, Telikani et al. [11] proposed a cost-sensitive deep learning model, which determines the cost function according to the cost matrix of data, so as to reduce the impact of dataset imbalance. He et al. [9] only used the large amount of benign traffic data to train the AE and judge whether the traffic is benign or malicious through the reconstruction rate of the AE. However, these kinds of models are complex and have poor adaptability to different traffic data.

From the perspective of data balancing, the traditional methods mainly include oversampling [12] and undersampling [13] techniques. Oversampling technology may cause over fitting problem, and undersampling technology will lead to insufficient learning of the data. Synthetic Minority Oversampling Technique (SMOTE) [14] technology is an improved algorithm based on oversampling. Instead of copying samples, it adds a small amount of noise to the samples to obtain different data. Qian et al. [15], Yan et al. [16] balanced the traffic datasets with the SMOTE algorithm and identified traffic on new datasets. However, SMOTE technology does not add new information to the samples. Goodfellow [17] first proposed generating sample data using the Generative Adversarial Network (GAN) in 2014. Different from the above methods, the data generated using the GAN contain data samples completely different from the original data.

Vu et al. [18] used an Auxiliary Classifier Generic Advantageous Network (ACGAN) [19] to expand traffic samples, balance SSH and non-SSH data, and then identified traffic. Dong et al. [8] used the Wasserstein GAN (WGAN) [20] to balance the traffic dataset and classify it. There are also studies that use the GAN and its derivative algorithms to generate traffic data [12,21,22], mixed with real data and train models and improving the performance of IDS and malware detection systems. In the research of using GAN and its derivative algorithms to expand traffic datasets, many of the studies use the original traffic data and convert it into images, and then expand the datasets and identify the traffic. However, the data used by many machine learning algorithms to train models and predict categories are feature data. If we synthesize original data or images, we also need to extract features later. Moreover, the storage and operation of images need a large cost.

In the research on the synthesis of the feature data of traffic data, Merino et al. [23] used a GAN to generate attack traffic in the NSL KDD99 dataset and balanced the dataset. Shahriar et al. [24] proposed a GAN-based Intrusion Detection System (G-IDS), which uses a GAN to generate imbalanced and missing data and improve the detection ability of the intrusion detection system. Their experiments were also trained and tested on the NSL KDD99 dataset. However, the NSL KDD99 dataset is too outdated, and the characteristics of network traffic are relatively simple and regular. It is no longer suitable for the current complex network environment. Huang et al. [25] proposed an Imbalanced Generative Adversarial Network Intrusion Detection System (IGAN-IDS) to perform data balancing and traffic category recognition on NSL KDD99, UNSW-NB15 and CIC-IDS2017 datasets. The recognition accuracy was improved compared with other machine learning algorithms. However, their algorithm did not fully consider the feature attributes of traffic data and cannot fully reflect the feature distribution of traffic data.

In the research on traffic data expansion using the GAN and its derivative algorithms, there are mainly two approaches: expanding original traffic data and expanding feature data. The expansion schemes of original traffic data need to store the synthetic samples after expansion and then extract and filter the features, so as to identify malicious traffic. These schemes require a lot of storage costs, and the calculation of images also requires a high cost. As for the expansion schemes of feature data, many datasets used in the schemes are outdated and have limited reference significance. Moreover, the existing research on the distribution of flow data features is insufficient.

The motivation of this paper is to use feature data for expansion, which meets the requirements of machine learning algorithm training and prediction. In this way, only feature data need to be saved during storage, while Pcap data and images do not, which greatly reduces the storage cost. In the subsequent model training process, feature extraction is not required again, which reduces the calculation cost. What is more, in order to better deal with the discrete and continuous variables in the features of traffic data, we use the CTGAN [26] model to generate data.

In the proposed CTTGAN scheme, after obtaining the original traffic data, we extract and filter the data to get characteristers, and then expand small class samples of data with the CTGAN algorithm. Considering the need of practical application, we only use the amplified data as the training set and the real data as the test set. The main contributions of this paper are as follows:

1. We proposed the CTTGAN scheme to expand the small category samples in the traffic datasets. After the expansion, all the indicators have been improved, and the effect is stable.
2. In the field of traffic data synthesizing, our research focuses on one-dimensional tabular feature data rather than image data, which are applicable to machine learning models and greatly reduce the storage and computing costs.
3. The scheme uses the CTGAN model, which can obtain better results when processing discrete variables and continuous variables in traffic data at the same time.

The structure of this paper is arranged as follows. In Section 2, we introduce the principle of GAN, especially the derivative algorithms of GAN in tabular data generation. In Section 3, we introduce the proposed Conditional Tabular Traffic GAN (CTTGAN) scheme in detail and present the scheme’s flow chart and algorithm. In Section 4, the experimental results and a comparative analysis are given. Finally, conclusions are drawn in Section 5.

## 2. Preliminaries

Using GAN to expand samples can generate new samples that did not exist before, which will not cause over-fitting problems and can reflect the characteristics of samples well. Many GAN-derived algorithms have been proposed according to their properties in the fields of images [27], music [28], natural language generation [29] and so on. The scheme proposed in this paper aims to expand traffic feature data to balance the dataset, that is, to expand the tabular data.

In this section, we introduce the implementation principle of GAN and its derivative algorithms in the field of tabular data generation.

### 2.1. GAN and Conditional GAN

**Figure 1:**
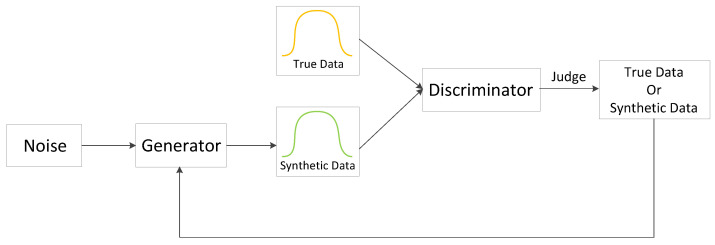
Schematic diagram of the GAN.

The basic idea of GAN [17] is to make the generator and discriminator confront each other to improve their performance. The schematic diagram is shown in Figure 1.

Let G(z) be the generator, and the input noise z∼p(z) be the output of synthetic data through G(z). The real data and the synthetic data are input to the discriminator D(x), and the discriminator outputs the discrimination result. The result of *D* is fed back to *G*, and *G* improves the generation algorithm to make the synthetic data closer to the real data. When more similar synthetic data and real data are input to *D*, *D* also needs to improve its discrimination ability to accurately distinguish the synthetic data from the real data. The above process is repeated continuously, and the generation and discrimination ability of *G* and *D* are continuously improved until the network reaches a Nash equilibrium. It can be considered that the data generated by the network are close to the real data. The objective function of the GAN is shown in Equation (1):

(1) $$\min_{G}\max_{D}V\left(D,G\right)=E_{x∼p_{\mathrm{data}\;}\left(x\right)}\left[\log D\left(x\right)\right]+E_{z∼P(z)}\left[\log\left(1-D\left(G\left(z\right)\right)\right)\right]$$

Mirza et al. [30] proposed that the GAN has the disadvantage that the modeling process is too free, which may make the training process difficult to control. In order to solve this problem, they proposed the Conditional GAN (CGAN). The idea of CGAN model is to add additional information variable *y* to the modeling of generator *G* and discriminator *D* to guide the generation of data. The objective function of CGAN is shown in Equation (2):

(2) $$\min_{G}\max_{D}V\left(D,G\right)=E_{x∼p_{\mathrm{data}\;}\left(x\right)}\left[\log D\left(x|y\right)\right]+E_{z∼P(z)}\left[\log\left(1-D\left(G\left(z|y\right)\right)\right)\right]$$

### 2.2. GAN in Generating Tabular Data

Many studies have been conducted on the generation of tabular data using the GAN. Yahi et al. [31] studied the use of the GAN to generate continuous laboratory time series data and proposed that it may be beneficial to combine the representation learning of the training queue before training the GAN model. Yu et al. [32] showed that it is difficult to pass the gradient update from the discriminator to the generator when using GAN to generate discrete tokens. They proposed the SeqGAN model, modeling the generator as stochastic, and directly updating the gradient of the generator. Choi et al. [33] proposed medGAN to generate realistic patient records. They focus on the generation of high-dimensional discrete variables (binary and count features). Lederrey et al. [34] proposed DATGAN model to generate population data. They combined expertise and deep learning methods and used directed acyclic graph to identify the relationships between variables.

### 2.3. Conditional Tabular GAN (CTGAN)

In the research of using GAN to generate tabular data, most of them are for discrete variables or continuous variables. When there are discrete variables and continuous variables in the real data at the same time, the algorithms will have difficulty generating data with the same distribution as the real data. To solve this problem, Xu et al. [26] proposed the CTGAN model. They designed a conditional generator to resample the imbalanced discrete columns. The reconstructed distribution of the real data is shown in Equation (3), in which $k^{*}$ represents the $i^{*}$th discrete column $D_{i^{*}}$ value:

(3) $$P\left(\;\mathrm{row}\;\right)=\sum_{k\in D_{i^{*}}}P_{G}\left(\;\mathrm{row}\;|D_{i*}=k^{*}\right)P\left(D_{i^{*}}=k\right)$$

## 3. Proposed Scheme

### 3.1. Design Concept

The network traffic characteristics include discrete variables such as the number of forward packets, the number of backward packets, the length of forward packets, the length of backward packets, etc., and continuous variables such as the number of forward packets per second, the number of backward packets per second, and the average packet length, etc. We propose the Conditional Tabular Traffic GAN (CTTGAN) scheme. In the stage of traffic sample expansion, the CTGAN model is used to expand each type of small sample to obtain the synthetic traffic data. In the CTGAN model, two fully connected hidden layers are used in both the generator and discriminator. The relu activation function is used in the generator and the leaky relu function is used in the generator.

### 3.2. Scheme Process

**Figure 2:**
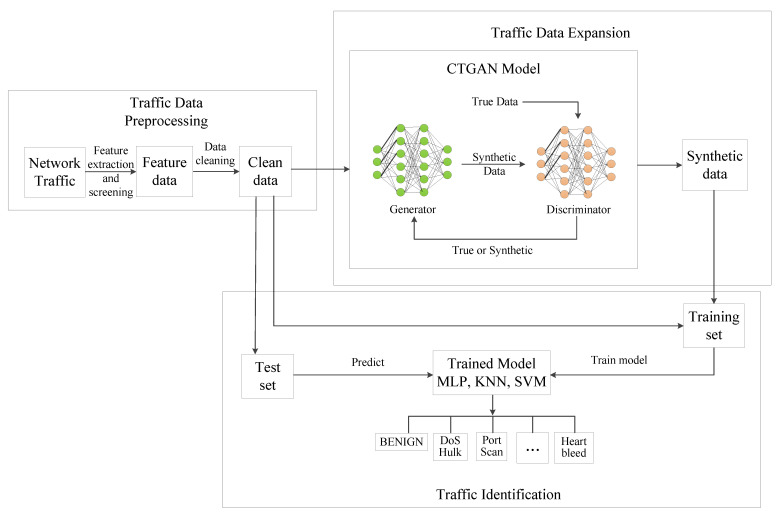
CTTGAN scheme flow chart.

The scheme flow chart is shown in Figure 2. First, preprocess the original traffic datasets to obtain the characteristic data. Next, expand each small traffic category to obtain the synthetic samples. Finally, train the identification model on the balanced dataset and make predictions.

### 3.3. Scheme Steps

**Figure 3:**
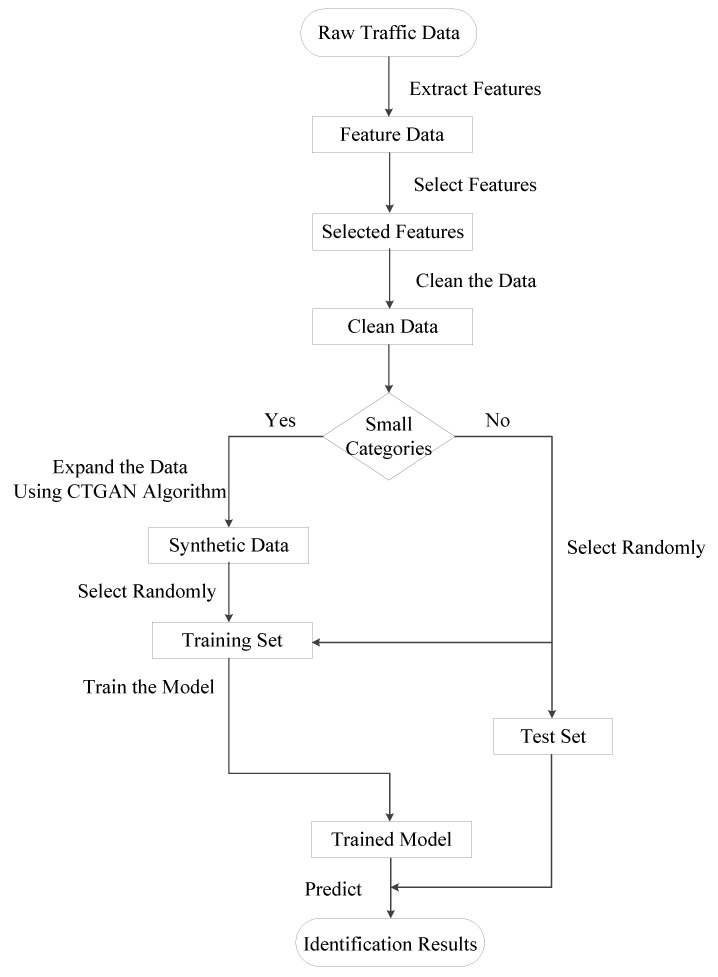
CTTGAN step flow chart.

In the data preprocessing section, first extract effective features of traffic data. Then, filter the features and remove unpractical ones, such as the timestamp, destination host and source host IP address. These features will make the traffic flow have obvious attributes; however, such features do not exist in practical applications. Next, we clean up the data, that is, remove data with missing terms and infinity values. In the small category traffic data expansion section, the CTGAN model is used to expand each small sample to obtain synthetic traffic data. Finally, the identification model is trained for traffic prediction. Considering the actual application demand, the traffic to be predicted shall be the real traffic. Therefore, we randomly selected part of the real traffic data as the test set, and mix the remaining real traffic data and synthetic traffic data as the training set to train the model. After the identification model is obtained, we predict the test set and obtain the results. The steps are shown in Algorithm 1 and Figure 3.
**Algorithm 1:** The Proposed CTTGAN
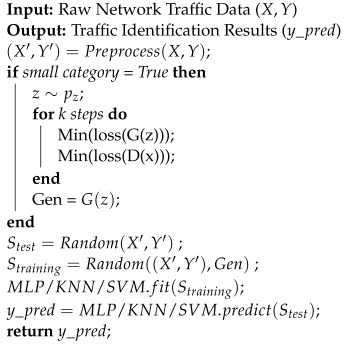


## 4. Experimental Results

In this section, we first introduce the dataset, evaluation indicators and experimental platform configuration, and then show the experimental results and conduct comparative analysis.

### 4.1. Dataset Description

We used CIC-IDS2017 dataset in the experiment, which was published by Canadian Institute of Network Security. The dataset collected network traffic data from 9 a.m. on 3 July 2017 to 5 p.m. on 7 July 2017, including benign traffic and 14 attack traffic events. The dataset is open and typical, and the traffic data are relatively new, which is consistent with the current network environment. The traffic category and quantity are shown in Table 1 and Table 2. It can be seen from Table 2 that the traffic data categories are imbalanced.

The dataset contains original network traffic data (PCAPs) and feature data (CSV) obtained by flow feature extraction tool CICFlowMeter. The feature data include 78 features such as flow duration, maximum packet length, minimum packet length, number of forward packets, number of reverse packets, etc. (the original feature data contains 79 features, in which the feature “forward packet header length” repeated twice and we deleted once). The following experiments were conducted with feature data.

It can be seen from Table 2 that the benign traffic accounts for more than 80 percent, far more than the sum of 14 types of malicious traffic. Among the 14 types of malicious traffic, 11 types of traffic samples, such as DoS GoldenEye and FTP-Patator, account for less than 1 percent, and three types of traffic samples, Infiltration, Web Attack Sql Injection and Heartbleed, account for less than 0.001 percent. That is, the traffic dataset is seriously imbalanced, which will make the machine learning algorithm biased towards the larger category samples, and the recognition rate of the smaller of category samples will be low.

### 4.2. Evaluation Indicators

We use three classical evaluation indicators, Recall, Precision and F1-score, in the experiment. The specific meanings are as follows:

TP (True Positive) indicates the number of positive cases recognized as positive, FP (False Positive) indicates the number of negative cases recognized as positive, FN (False Negative) indicates the number of positive cases recognized as negative and TN (True Negative) indicates the number of negative cases recognized as negative. In the case of multi-classification problems, when evaluating the classification of one category, the samples of this category are recorded as positive cases, and all the other samples are recorded as negative cases.

(4) $$\;\mathrm{Recall}\;=\frac{TP}{TP+FN}$$

(5) $$\;\mathrm{Precision}\;=\frac{TP}{TP+FP}$$

(6) $$F1-\mathrm{score}=\frac{2\times \;\mathrm{Recall}\;\times \;\mathrm{Precision}\;}{\;\mathrm{Recall}\;+\;\mathrm{Precision}\;}$$

Recall reflects the ratio that a certain type of data are correctly detected; Precision reflects the ratio of all data detected as a certain type of data; F1-score takes both recall and precision into account. For a good traffic detection model, it should have high Recall, Precision and F1-score.

### 4.3. Experimental Platform Configuration

The experiments were conducted on Windows 11 64-bit OS and 16 GB of RAM. The code was written in Python 3.8 using the sklearn 0.24.1, sdv 0.14.0, pandas 1.2.4, numpy 1.20.1 and matplotlib 3.5.1 libraries. We called some algorithms in the sklearn library to segment the training set and test set, draw the confusion matrix and train the MLP, KNN and SVM models; the Sdv library was used to train the CTGAN model and generate data; the pandas and numpy libraries were used to preprocess data; and the Matplotlib library was used to draw and save pictures. The download website, brief introduction and used functions of the libraries are shown in Table 3. The IDE used in the experiment is pycharm, version 2020.3 x64.

### 4.4. Experimental Results and Analysis

#### 4.4.1. Identification Results of Raw Data

In Experiment 1, we extracted the categories with data quantities greater than 10,000. For DDoS, DOS Hulk, PortScan and DoS GoldenEye (data quantities between 10,000 and 1,000,000), we extracted 10,000 pieces of data. Subsequent experiments can verify that 10,000 pieces of data are enough to stabilize the model recognition rate. For BENIGN data (with a data quantity of more than 1,000,000), considering that the normal network samples contain many types of traffic, such as accessing normal web pages, sending and receiving emails, downloading data, etc., in order to fully characterize benign traffic, 100,000 samples were selected for experiments. The quantity of the data used in the experiment one is shown in Table 4.

**Figure 4:**
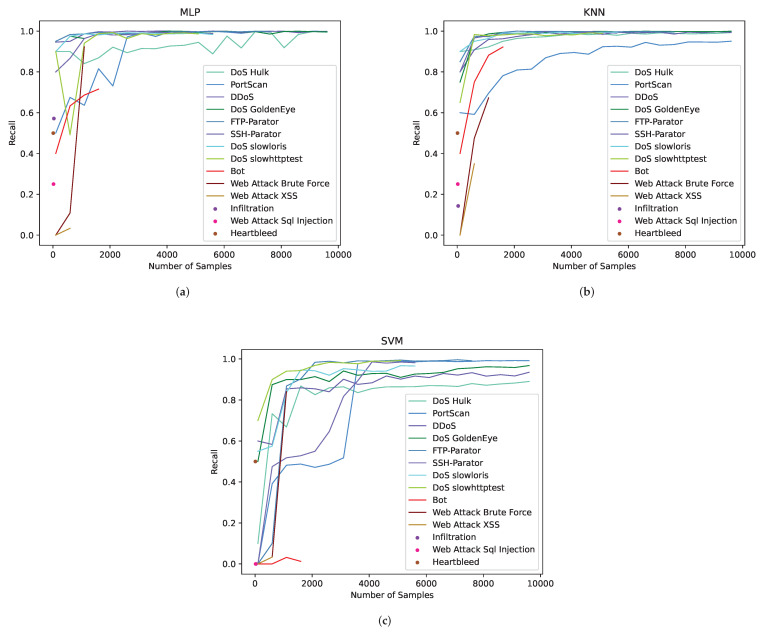
Growth curve of Recall indicator with number of traffic samples using three machine learning algorithms. (**a**) MLP; (**b**) KNN; (**c**) SVM.

We use MLP, KNN and SVM machine learning algorithms to classify the raw imbalanced data. For the 14 categories of malicious traffic, we increase the number of training samples step by step, and obtain the growth curve of the Recall indicator with the number of samples, as shown in Figure 4. In order to reflect the relationship between the Recall indicator of each traffic category and data quantity, experiments are carried out for each traffic category; that is, for each category, the number of samples is increased step by step, the data of other categories are kept unchanged and the change in the Recall value of this category is recorded.

There are 14 curves in Figure 4a–c representing the change in the Recall values of 14 traffic categories with the number of samples. In the three figures, there are six traffic categories in which the Recall values are not stable. They are Bot, Web Attack Brute Force, Web Attack XSS, Infiltration, Web Attack Sql Injection and Heartbleed. The experimental results show that in different machine learning algorithms, all kinds of traffic samples need to reach a certain amount of data to make the trained model stable.

As for the selection of data volume, we give some supplementary explanations. The quantity of the extracted data is related to many factors, such as the complexity of the data itself, the number of extracted features, the significance of the features, whether the extracted features are reasonable and so on. In addition, the selection of data volume is also closely related to the architecture and implementation functions of the machine learning model. It can be seen from the experimental results that when the sample size of the original traffic data is sufficient, 5000 pieces of data can be used to train different machine learning models to achieve stability. This data volume may be of great reference value for datasets similar to the CIC-IDS2017 dataset (78 features and 14 traffic categories).

#### 4.4.2. Identification Results after CTTGAN Expansion

**Figure 5:**
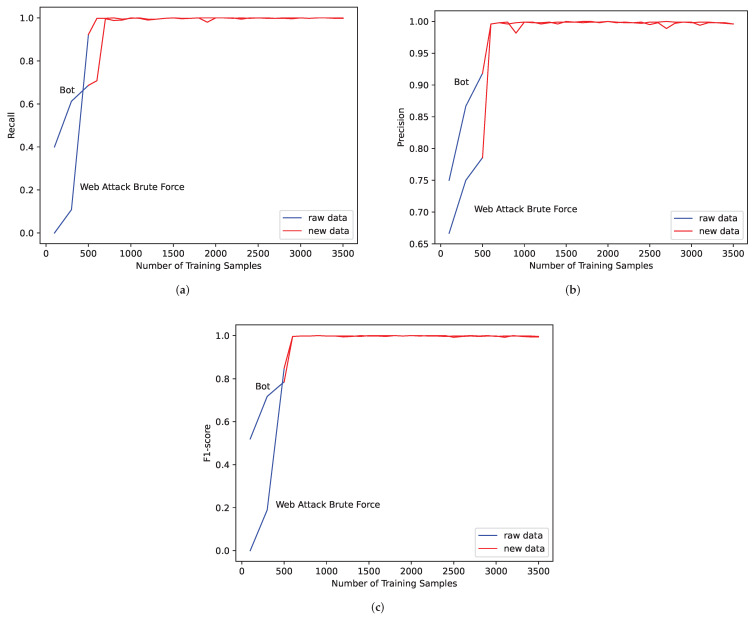
Experimental results of Bot and Web Attack Brute Force in MLP identification algorithm. (**a**) Recall; (**b**) Precision; (**c**) F1-score.

For the six traffic categories with insufficient data in experiment one, we consider the quantity of Web Attack XSS, Infiltration, Web Attack Sql Injection and Heartbleed are too small to fully reflect the characteristics of the samples, so these four traffic categories will not be considered in subsequent studies. For Bot and Web Attack Brute Force, we use the CTGAN algorithm to expand them and conduct identification experiments. The following experiments use MLP algorithm for identification. The values of Recall, Precision and F1-score are obtained as shown in Figure 5.

Blue curves in the figures represent the original data, and red curves represent synthetic data. Considering the needs of practical application, in the experiments of synthetic data, 500 pieces of real traffic data are randomly selected as the test set, and another 500 real traffic data are randomly selected and are mixed with synthetic data as the training set. The control variable method is used in the experiment; that is, when changing the quantity of Bot traffic data, the other categories of data are kept unchanged, and we record the indicators of Bot data. The same operation is performed on Web Attack Brute Force data. It can be seen from the experimental results that the indicators of Bot and Web Attack Brute Force have improved after expansion and finally reach stability.

**Figure 6:**
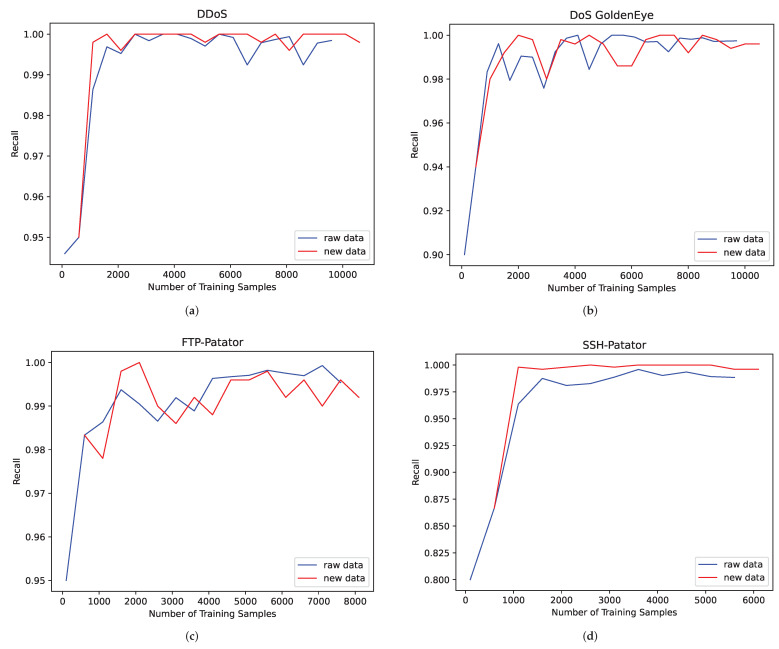
Experimental results of four traffic categories with sufficient sample sizes in the MLP identification algorithm. (**a**) DDoS; (**b**) DoS GoldenEye; (**c**) FTP-Patator; (**d**) SSH-Patator.

To verify the effectiveness of the proposed scheme, we selected traffic categories with sufficient sample sizes to perform a verification with. DDoS, DoS GoldenEye, FTP-Patator and SSH-Patator are selected, and the results are shown in Figure 6.

The experimental results show that the synthetic samples have a similar fluctuation trend with the original samples, which indicates that the synthetic samples can reflect the characteristics of the original data well. In addition, in order to verify the effectiveness of the proposed scheme, we use the MLP, KNN and SVM algorithms to identify the traffic of the expanded dataset. The results are shown in Table 5. For Bot and Web Attack Brute Force, 500 real samples are randomly selected as the test set, and 4500 generated samples are used as the training set. The data volume of other traffic categories is the same as that of Experiment 1.

In the recognition results obtained by the KNN, SVM and MLP algorithms, the recognition recall index of Bot and Web Attack Brute Force all reaches more than 0.99, and all training sets are real samples. The results show that the proposed scheme is effective.

#### 4.4.3. Comparative Experiments

**Figure 7:**
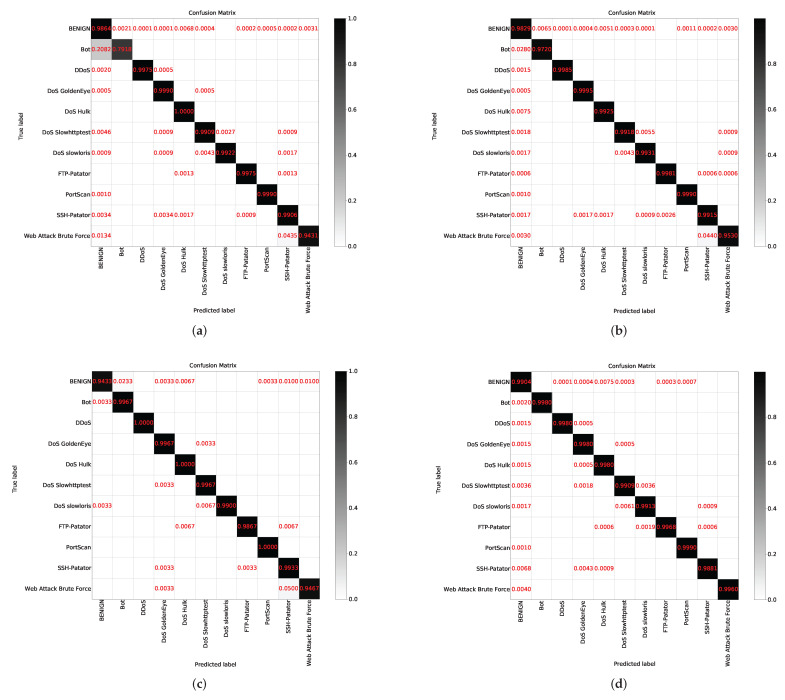
Confusion matrix of comparative experiment. (**a**) Original data; (**b**) Oversampling; (**c**) Undersampling; (**d**) CTTGAN.

The above experiments verify the effectiveness of the proposed scheme. Next, we further compare the CTTGAN scheme with oversampling and undersampling, the two most common schemes used to balance datasets in machine learning algorithms [35]. Oversampling refers to the repeated sampling of a few samples, and undersampling refers to the discarding of some large samples to achieve a balance between the data. The results of the MLP algorithm are shown in Table 6. The quantity of data for the traffic categories is shown in brackets. The Recall values of the small traffic categories in the CTTGAN scheme reach more than 0.99, which are marked in red. The confusion matrix of the experimental results is shown in Figure 7.

In Experiments 1 and 2, it can be concluded that both real samples and synthetic samples are stable when the quantity reaches 5000. Therefore, in the oversampling experiment, we repeatedly sampled Bot and Web attack brute force samples to 5000, and the data of other traffic categories remain unchanged. In the undersampling experiment, the quantity of Web attack brute force is 1497, and we randomly selected 1500 pieces of data for other traffic categories to balance the data. In the CTTGAN experiment, the Bot and Web attack brute force samples were expanded to 5000. The data of other traffic categories remained unchanged.

The results show that the recognition rate of the Bot and Web Attack Brute Force samples is low in the original case. In the oversampling experiment, the recognition rate of Bot has been greatly improved, and the recognition rate of Web Attack Brute Force has been slightly improved. In the undersampling experiment, the recognition rate of Bot reaches more than 0.99, the recognition rate of Web Attack Brute Force is almost unchanged and the BENIGN recognition rate decreases. In the CTTGAN experiment, the recognition rate of each category is high.

#### 4.4.4. Discussion and Analysis

In the experiments, we synthesize feature data of network traffic, rather than original data or image. The synthetic data can be directly input to machine learning algorithms, saving storage costs and computing costs. In addition, it may be more reasonable to calculate and process the feature data in the CTTGAN model. In some schemes that convert network traffic into images, the first n bytes of the original network traffic are converted into gray images. These bytes contain the destination IP, source IP and other information, which is unreasonable to be used to identify the traffic category. Such problems can be solved in the CTTGAN.

The network traffic features include both continuous variables and discrete variables. We use the CTGAN algorithm to generate two types of data at the same time. The synthetic data have similar distributions to the original data. First, we conduct experiments to verify that in different identification algorithms, the traffic data need to reach a certain quantity to make the model achieve a stable recognition rate. Secondly, we verify that for small category traffic samples, the synthetic data can improve the performance of the model. For large category samples, the synthetic data have similar fluctuation trends to the original one. These experimental results prove that the data generated by the CTTGAN scheme are close to the real data, and the generated data can be used as a supplement to the insufficient samples. What is more, we perform traffic recognition for all categories. In the MLP, KNN and SVM algorithms, the recognition rate of the expanded samples reaches more than 0.99. Finally, we compare the CTTGAN model with oversampling and undersampling schemes. The performance of the CTTGAN is better than the oversampling and undersampling schemes on the whole, which proves that the proposed CTTGAN scheme is effective and has practical significance.

In the experiments, all test sets are composed of real data, which proves that the synthetic data can be used as a supplement in the training of the model to improve its performance in real detection scenarios.

## 5. Conclusions

In this paper, we propose the CTTGAN model to expand network traffic samples to balance the dataset in order to improve the recognition rate of machine learning algorithms. Different from most traffic data expansion models, the CTTGAN model does not convert network traffic data into images, but extracts its effective features and then expands the feature data. In this way, the synthetic feature data conform to the data structure of machine learning algorithms, and we do not need to extract features after data expansion. This reduces storage costs and computational complexity and speeds up computing. Experiments show that the recognition rate of traffic categories with less data is low, and the recognition rate increases and reaches a stable level when there is sufficient data. After expanding the small category samples with the CTTGAN model, the recognition rate reaches more than 0.99, and the model has good stability. We also use the CTTGAN to synthesize the large category samples to verify that the fluctuation trend is similar to the real data. Therefore, the proposed CTTGAN model is effective. Moreover, the recognition rate of the CTTGAN model is higher than that of the oversampling and undersampling schemes, which proves that the CTTGAN model has good experimental results and practical value.

In future work, we will further study the expansion of categories with too few samples, such as Web Attack XSS, Infiltration, Web Attack Sql Injection and Heartbleed. We will study how to fully mine the characteristics of these categories of data, reflect the overall distribution and then realize reasonable expansion of these data to achieve effective identification. In addition, we consider the study of instant identification of malicious traffic, which is of great significance for practical applications.

## Figures and Tables

**Table 1:** Overview of dataset CIC-IDS2017.

Date	Traffic Category
Monday	BENIGN
Tuesday	BENIGN, FTP-Parator, SSH-Parator
Wednesday	BENIGN, DoS Hulk, DoS GoldenEye, DoS slowloris, DoS slowhttptest, Heartbleed
Thursday	BENIGN, Web Attack Brute Force, Web Attack XSS, Web Attack Sql Injection, Infiltration
Friday	BENIGN, PortScan, DDoS, Bot

**Table 2:** The categories and quantities of dataset CIC-IDS2017.

Traffic Category	Quantity	Proportion
BENIGN	2,260,360	80.33%
DoS Hulk	229,198	8.15%
PortScan	157,703	5.60%
DDoS	127,082	4.52%
DoS GoldenEye	10,289	0.37%
FTP-Patator	7894	0.28%
SSH-Patator	5861	0.21%
DoS slowloris	5771	0.21%
DoS slowhttptest	5485	0.19%
Bot	1943	0.07%
Web Attack Brute Force	1497	0.05%
Web Attack XSS	648	0.02%
Infiltration	34	0.0012%
Web Attack Sql Injection	21	0.0007%
Heartbleed	11	0.0004%

**Table 3:** The download website, description and used functions of the libraries (all web links accessed on 5 June 2022).

Library	Download Website	Description	Used Function
sklearn	https://scikit-learn.org	Tools for predictive data analysis	confusion_matrix, train_test_split, preprocessing, MLPClassifier, KNeighborsClassifier, SVC
sdv	https://github.com/sdv-dev/SDV	A synthetic data generation ecosystem	CTGAN, evaluate
pandas	https://pandas.pydata.org	A data analysis and manipulation tool	read_csv, factorize, DataFrame
numpy	https://numpy.org	A scientific computing package	diag, sum, mean
matplotlib	https://matplotlib.org	A comprehensive visualization library	pyplot

**Table 4:** Data quantity in experiment one.

Traffic Category	Quantity
BENIGN	100,000
DoS Hulk	10,000
PortScan	10,000
DDoS	10,000
DoS GoldenEye	10,000
FTP-Patator	7894
SSH-Patator	5861
DoS slowloris	5771
DoS slowhttptest	5485
Bot	1943
Web Attack Brute Force	1497
Web Attack XSS	648
Infiltration	34
Web Attack Sql Injection	21
Heartbleed	11

**Table 5:** Identification results of the expanded dataset.

Data Category	Recall		
	MLP	KNN	SVM
BENIGN	0.9904	0.9881	0.9682
DoS Hulk	0.9980	0.9925	0.9015
PortScan	0.9990	0.9590	0.9910
DDoS	0.9980	0.9940	0.9350
DoS GoldenEye	0.9980	0.9975	0.9730
FTP-Patator	0.9968	0.9987	0.9899
SSH-Patator	0.9981	0.9949	0.9889
DoS slowloris	0.9913	0.9931	0.9671
DoS slowhttptest	0.9909	0.9918	0.9854
Bot	0.9980	0.9960	0.9980
Web Attack Brute Force	0.9960	0.9960	1.0000

Note: Red indicates the Recall index of small category samples in CTTGAN scheme, all of which are above 0.99.

**Table 6:** Experimental results of the comparative experiment.

Data Category	Recall			
	Raw Data (Amount)	Over Sampling (Amount)	Under Sampling (Amount)	CTTGAN (Amount)
BENIGN	0.9864 (100,000)	0.9829 (100,000)	0.9433 (1500)	0.9904 (100,000)
DoS Hulk	1.0000 (10,000)	0.9925 (10,000)	1.0000 (1500)	0.9980 (10,000)
PortScan	0.9990 (10,000)	0.9990 (10,000)	1.0000 (1500)	0.9990 (10,000)
DDoS	0.9975 (10,000)	0.9985 (10,000)	1.0000 (1500)	0.9980 (10,000)
DoS GoldenEye	0.9990 (10,000)	0.9995 (10,000)	0.9967 (1500)	0.9980 (10,000)
FTP-Patator	0.9975 (7894)	0.9981 (7894)	0.9867 (1500)	0.9968 (7894)
SSH-Patator	0.9906 (5861)	0.9915 (5861)	0.9933 (1500)	0.9881 (5861)
DoS slowloris	0.9922 (5771)	0.9931 (5771)	0.9900 (1500)	0.9913 (5771)
DoS slowhttptest	0.9909 (5485)	0.9918 (5485)	0.9967 (1500)	0.9909 (5485)
Bot	0.7918 (1943)	0.9720 (5000)	0.9967 (1500)	0.9980 (5000)
Web Attack Brute Force	0.9431 (1497)	0.9530 (5000)	0.9467 (1497)	0.9960 (5000)

Note: Red indicates the Recall index of small category samples in the CTTGAN scheme, all of which are above 0.99.

## Data Availability

The network traffic dataset CIC-IDS2017 can be downloaded from website: https://www.unb.ca/cic/datasets/ids-2017.html (accessed on 5 June 2022).
